# Treatment outcome of chronic low back pain and radiographic lumbar disc degeneration are associated with inflammatory and matrix degrading gene variants: a prospective genetic association study

**DOI:** 10.1186/1471-2474-14-105

**Published:** 2013-03-22

**Authors:** Ahmad Omair, Marit Holden, Benedicte Alexandra Lie, Olav Reikeras, Jens Ivar Brox

**Affiliations:** 1 Department of Orthopaedics, Oslo University Hospital-Rikshospitalet, Sognsvannsveien 20, Oslo 0027, Norway; 2 Department of Medical Genetics, University of Oslo and Oslo University Hospital-Ullevål, Oslo, Norway; 3 Norwegian Computing Center, Blindern, Oslo, Norway

## Abstract

**Background:**

Inflammatory and matrix degrading gene variants have been reported to be associated with disc degeneration. Some of these variants also modulate peripheral pain. This study examines the association of these genetic variants with radiographic lumbar disc degeneration and changes in pain and disability at long-term after surgical and cognitive behavioural management.

**Methods:**

93 unrelated patients with chronic low back pain (CLBP) for duration of >1 year and lumbar disc degeneration were treated with lumbar fusion or cognitive intervention and exercises. Standardised questionnaires included the Oswestry Disability Index (ODI) and Visual Analog Score (VAS) for CLBP, were filled in by patients both at baseline and at 9 years follow-up. Degenerative changes at baseline Magnetic Resonance Imaging and Computed Tomography scans, were graded as moderate and severe (N=79). Yield and quality of blood and saliva DNA was assessed by nano drop spectrophotometry. Eight SNPs in 5 inflammatory and matrix degrading genes were successfully genotyped. Single marker and haplotype association with severity of degeneration, number of discs involved, changes in ODI and VAS CLBP, was done using Haploview, linear regression and R-package Haplostats.

**Results:**

Association analysis of individual SNPs revealed association of *IL18RAP* polymorphism rs1420100 with severe degeneration (*p* = 0.05) and more than one degenerated disc (*p* = 0.02). From the same gene two SNPs, rs917997 and rs1420106, were found to be in strong linkage disequilibrium (LD) and were associated with post treatment improvement in disability (*p* = 0.02). Haplotype association analysis of 5 SNPs spanning across *IL18RAP*, *IL18R1* and *IL1A* genes revealed significant associations with improvement in disability (*p*=0.02) and reduction in pain (*p*=0.04). An association was found between *MMP3* polymorphism rs72520913 and improvement in pain (*p* = 0.03) and with severe degeneration (*p* = 0.006).

**Conclusions:**

The findings of the current study suggest a role of variation at inflammatory and matrix degrading genes with severity of lumbar disc degeneration, pain and disability.

## Background

Fifty percent report to have back pain yearly in Norway [1]. Degeneration of the lumbar intervertebral disc is regarded as a common cause of chronic low back pain (CLBP) [2]. The etiology of lumbar disc degeneration (LDD) is complex and not fully explained. LDD is considered to be a multifactorial disorder involving numerous genetic and environmental factors and their interactions. Heavy physical loading, trauma, bending, twisting, prolonged non-neutral work postures, vibrations during vehicle driving, cigarette smoking have been suggested to be associated with disc degeneration [3,4]. The pathogenesis of LDD involves the degradation of the normal disc matrix into a disorganized fibrous and less cartilaginous disc, progressing to the formation of clefts and fissures and ultimately compromising the disc integrity [5]. Magnetic Resonance Imaging (MRI) studies have established a strong heritability of degenerative changes among twins [6] and genes related to the structural proteins of the discs (aggrecan and collagens), inflammation and matrix degradation have been reported to be associated with the pathologic and radiographic changes of LDD [7-10].

The correlation between severity of degeneration and clinical symptoms is not strong and recent studies reported no correlation between changes in degenerated disc and changes in treatment [11,12].Variants of some genes coding for inflammatory mediators: interleukin 1A (IL1A); interleukin 18 receptor 1 (IL18R1); interleukin 18 receptor accessory protein (IL18RAP); cyclooxygenase 2 (COX2) and matrix metalloprotease 3 (MMP3), have all been found to be associated with different phenotypes of LDD [9,10,13-16]. They have also been reported to be involved in peripheral modulation of pain [17]. These genes are candidates for assessment of their association with degeneration, pain and disability.

Pro inflammatory cytokines are involved in extracellular matrix degradation and fibrosis of the disc, and play a role in peripheral neuropathic pain. IL1 causes degeneration by regulating the destruction of disc matrix by activating degradative enzymes and inhibiting resynthesis of proteoglycans. It also modulates pain by inducing prostaglandin E2 (PGE2) synthesis which in turn enhances the activity of phospholipase A2 and COX2. IL1 secreted by herniated discs, along with IL6 and TNF-α sensitize the nociceptors that innervate the disc [18,19]. IL18 is another proinflammatory cytokine that shares many biological, structural and functional properties with IL1 [20] and mediates its function through its receptor IL18R (IL18R1 and IL18RAP). It exerts its pro-inflammatory effects by inducing the production of IL-1β, TNF-α, chemokines, nitric oxide and prostaglandins [21,22].

MMP3 (stromelysin 1) mediates degeneration of the disc by degradation of matrix proteoglycans and collagens [23] and also contributes to neuropathic pain after peripheral nerve injury [24]. *MMP3* gene polymorphism *5A/6A* (rs72520913) regulates the *MMP3* gene expression with *5A* allele possessing twice the promoter activity than *6A*[25].

The COX2 gene (PTGS2) may be involved in disc herniation through the up regulation of PGE2 [26]. It is also reported to be involved in the peripheral modulation of pain [17,27].

Associations between inflammatory and matrix degrading gene variants and changes in long- term pain and disability after surgical and cognitive behavioural management, have to our knowledge not been investigated previously. The aim of the present study was to examine these associations. We also analyzed the yield and purity of DNA acquired from both blood and saliva in our patients, in light of an increase in use of saliva as a source of genomic DNA for genetic association studies, compared to the blood [28].

## Methods

### Study design

The cohort was from a randomized control trial, and a prospective design was used for obtaining clinical data at long term follow-up and was in the current study used for genetic association analyses.

### Patient sample

124 unrelated Norwegian patients with chronic LBP recruited from two previously published randomized control trials [29-32] were invited to participate. At baseline each patient was examined by at least one spine surgeon and one specialist in physical and rehabilitation medicine. The inclusion criteria were: age 25-60 years; LBP with duration >1 year despite of undergoing supervised non-operative treatment; Oswestry disability index (ODI) score > 30 of 100 points; and disc degeneration at one or two levels i.e. L4-L5 and/or L5-S1 as assessed by plain radiography. Exclusion criteria were: widespread myofascial pain; spinal stenosis with reduced walking distance and associated neurological signs; recurrent disc herniation or lateral recess stenosis with clinical signs of rediculopathy; inflammatory disease; previous spinal fracture; previous surgical fusion of spine; pelvic pain; generalized disc degeneration on plain radiographic assessment; ongoing serious somatic or psychiatric disease that could exclude treatment alternatives; registered medical abuse and reluctance in accepting either one or both treatment modalities of this study.

Ninety nine patients responded to the follow up examination at median 9 years (range 7-11 years). Ninety three patients (35 males and 58 females) were ethnically West European, and hence 6 were excluded due to a non-European ethnicity. Out of the 93 patients, 51 were randomized to lumbar instrumented postero-lateral fusion with transpendicular screws at L4-L5 and /or L5-S1 and 42 to cognitive intervention and exercises given by physiotherapists and a specialist in physical and rehabilitation medicine. This intervention involved one week plus two weeks at outpatient facility interrupted by two weeks at home. Five patients randomized to fusion did not undergo surgery and 14 patients randomized to cognitive treatment had later undergone fusion.

The eligible patients were informed orally as well as in writing about the study procedures before consenting. The Regional Committee for Medical Research Ethics in Health Region South-East Norway approved the study. It was also recommended by the Biobank Register at Norwegian Institute of Public Health and by the patient’s ombudsman at, Oslo University hospital.

### Outcome variables of pain and disability

The clinical data regarding age, ethnicity, gender, and presence of any other medical condition was obtained at baseline. At baseline and at 9 year follow-up, these patients were given standardised questionnaires for assessing the disability and pain. Disability was estimated by ODI comprising 10 questions about pain related disability, each having six verbal response alternatives. The sum of the response score was calculated and presented as percentage where 0% represents no pain and disability, and 100% represents the worst possible pain and disability [33]. LBP intensity was scored on three vertical visual analog scales (VAS), ranging from 0 (no pain) to 100 (worst pain imaginable). Patients scored their maximum pain, minimum pain and current pain the last week, respectively. The mean of these three measurements was calculated [34].

The outcome variables were the change in ODI and VAS scores at follow-up (post treatment value – pre treatment value).

### Radiological assessment of disc degeneration

Disc degeneration in all patients was assessed for levels L1-S1 by plain radiography. MRI (*N* = 65) and Computed Tomography scanning [CT] (*N* = 14) were also used, but not in all patients. MRI signal changes in lumbar discs were graded into six patterns [35]. Pattern 1: Thickened cleft but normal disc signal intensity and height with no protrusion; Pattern 2: Similar to pattern 1 with slight overall reduction in disc signal intensity; Pattern 3: Normal disc signal intensity, height and cleft with small protrusion; Pattern 4: Disc signal intensity was further reduced, mild narrowing of the disc space, cleft present with definite protrusion of the disc; Pattern 5: Marked reduction in disc signal intensity and height with protrusion and cleft poorly defined; Pattern 6: Minimal or lost disc signal with marked resorption and protrusion of the disc with no cleft.

MRI changes were dichotomized into moderate if assessed to be of pattern 1-4; and severe if the pattern was 5 or 6. Degeneration on CT scans was classified moderate if disc height was considered to be < 50% of the normal height and severe if disc height was reduced > 50%.

### DNA extraction

Genomic DNA from 89 patients was extracted from approx 9 ml of venous blood by salting out method [36]. From all 93 patients, DNA was extracted from approx 2 ml of saliva using a collection kit, purifier and protocol from DNA genotek (Kanata, Ontario Canada). This means that in four patients DNA was extracted from saliva only. Both blood samples and saliva were collected at the Hospital facility. DNA quality and concentration were measured on a DNA spectrophotometer (ND-1000, NanoDrop, Wilmington USA).

**Figure 1:**
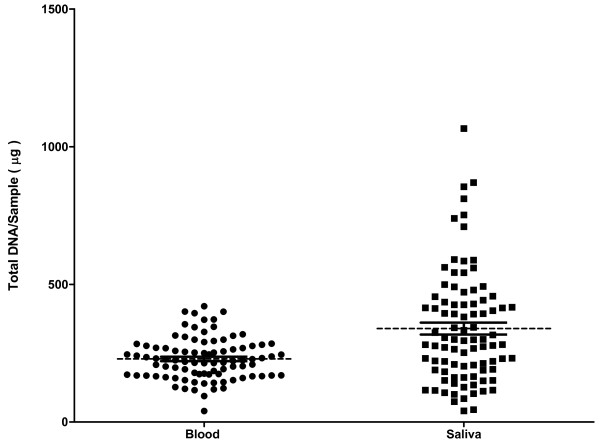
**Scattergram of DNA yield (*μg*) in 89 blood and saliva samples.** Scattergram of total DNA yield (*μg*) in 89 blood and saliva samples. The dashed line represents the mean value and continuous lines denote standard error of mean (SEM).

For the 89 patients, we compared the purity and total DNA yield (*μg*) from blood and saliva samples. The amount of DNA (*μg*) obtained given as mean [SEM] from blood sample was 229.3 [8.0] and from saliva, it was 339.3 [21.6] respectively (Figure 1). Purity of the DNA was assessed by the ratio of absorbance at 260 nm and 280 nm (A260/280), and values ≥ 1.8 indicate a pure DNA. The mean A260/280 ratio for the blood sample was 1.86 [0.001] and 1.94 [0.007] for saliva, respectively. There were no drop out for DNA samples extracted from blood, but one drop out from a DNA sample extracted from saliva (A260/280 < 1.8). For 48 patients, DNA from blood as well as saliva was genotyped together, and genotype success rate of DNA from both sources was comparable for the tested SNPs.

### Selection of single-nucleotide polymorphisms and genotyping

A total of eight single-nucleotide polymorphisms (SNPs) from five different inflammatory and matrix degrading genes were tested on the basis of previously reported associations with disc degeneration and their established role in pain related pathways. These included SNPs from interleukin genes (*IL1A, IL18R1, and IL18RAP*), matrix metalloprotease-3 gene (*MMP3*), and cyclooxygenase-2 gene (*COX2*).

Genotyping for the SNPs was performed by Sequenom^™^ system, using matrix-assisted laser desorption/ionization time-of-flight (MALDI-TOF) mass spectrometry at the Centre for interactive genetics; Cigene, Norwegian University of Life Sciences (UMB) Ås, Norway.

### Statistical analysis

Hardy-Weinberg equilibrium, pair wise linkage disequilibrium (LD), genotype success rate and minor allele frequency were calculated using the statistical programme Haploview version 4.2 [37]. The cut off value for divergence from Hardy-Weinberg equilibrium was (p ≥ 0.001).

Linear and logistic regression analysis was performed for assessing the effect of each individual SNP on the regression of degeneration, number of segments involved, ODI and LBP change as dependent variables, along with controlling for the covariates age, sex and fusion. Association was tested using three different types of genetic models on the same data set. A recessive genetic model, assuming that severe degeneration and change in post treatment pain scores was increased / decreased when having two copies of a specific allele compared to having only one or zero copies of the specific allele. The mean difference in pain and disability score (*β*) between patients having two copies and those having one or zero copies was estimated in regression analysis. In the additive model, we assumed that the change in the progression and pain scores was increased two fold by possessing two copies of the specific allele, compared to having a single copy. For the dominant genetic model we pre specified the dominant allele to be a risk allele and carriers were compared with the opposite non-risk homozygous alleles [38]. A *p*-value ≤ 0.05 was considered statistically significant. We consider our study to be an explorative genetic association study, and therefore the p-values were not formally corrected for multiple testing.

Association between the common haplotypes of the SNPs with frequency > 0.02 and severity of degeneration, numbers of segments involved, pain and disability, along with effect of the covariates was tested using the R package Haplostats [39]. The estimated frequencies and regression coefficients were computed by the function haplo.glm using an additive model. The reference haplotype was selected to be the most frequent haplotype as a baseline for linear regression by the software.

## Results

The genotype success rates for the eight SNPs were 100% and minor allele frequencies observed for the tested SNPs in the present population were comparable to what has been reported for European Hap Map samples (Table 1). No divergence from Hardy-Weinberg equilibrium observed for any of the tested markers.

**Table 1:** Overview of the selected SNPs and their genotyping performance

SNP*	**Gene**	HWE** *p*-value	**GRS %†**	**Alleles‡**	**MAF§ cases**	**MAF ∞ reported**
rs2071375	*IL1A*	1.0	100	*T/C*	0.25	0.21
rs2287037	*IL18R1*	0.73	100	*T/C*	0.32	0.39
rs1420100	*IL18RAP*	1.0	100	*A/C*	0.50	0.51
rs1420106	*IL18RAP*	0.7	100	*A/G*	0.25	0.21
rs917997	*IL18RAP*	1.0	100	*T/C*	0.27	0.20
rs5277	*COX2*	0.81	100	*G/C*	0.19	0.21
rs4140564	*COX2*	1.0	100	*G/A*	0.05	0.08
rs72520913	*MMP3*	0.09	100	*A/C*	0.50	Not reported

* Single nucleotide polymorphism; ** Hardy-Weinberg equilibrium; **†** Genotype success rate;

**‡** Minor/Major alleles; **§** Minor allele frequency; **∞** MAF HapMap-CEU European.

### SNP associations with pain and disability outcome

Association analysis of individual SNPs with the 9 years post treatment change in pain and disability scores using a recessive genetic model among 93 patients, revealed statistically significant associations between post treatment improvement in disability (ODI change) and two SNPs from *IL18RAP* gene, rs1420106 and rs917997 (*p* = 0.02) (Table 2). The alleles at these two polymorphisms rs1420106-*A* and rs917997-*T* were in strong LD (*D'*=1 and *r*^*2*^=0.9) and hence represented the same association. A significant association (*p* = 0.03) was also observed between post treatment improvement in pain and a polymorphism (rs72520913) of the *MMP3* gene (Table 2). Our study had 80% power to detect a change of 22.2 in ODI and 19.7 in VAS LBP with a significance level of 0.05 for the associated single nucleotide polymorphisms (SNPs).

**Table 2:** Association analysis of inflammatory and matrix degrading gene polymorphisms with 9 years ODI change and VAS LBP change in 93 patients with LDD and LBP

			**ODI change**		**VAS LBP change**	
SNP*	**Gene**	**Recessive genotype**	**Mean difference (*β*) † (95% CI)∞**	***p *value**	**Mean difference (*β*) (95% CI)**	***p *value**
rs2071375	*IL1A*	*T/T*	8.8 (-8.3 - 26)	0.31	11.3 (-11.3 - 33.9)	0.32
rs2287037	*IL18R1*	*T/T*	6.8 (-8.6 – 22.2)	0.38	11.0 (-9.3 - 11)	0.29
rs1420100	*IL18RAP*	*A/A*	6.1 (-4 – 16.2)	0.23	5.1 (-8.3 – 18.5)	0.45
rs1420106	*IL18RAP*	*A/A*	19.4 (3.9 - 35)	0.02	18.4 (-2.5 – 39.3)	0.08
rs917997	*IL18RAP*	*T/T*	19.4 (3.9 - 35)	0.02	18.4 (-2.5 – 39.3)	0.08
rs5277	*COX2§*	*G/G*	15.0 (-5.8 – 35.8)	0.16	24.4 (-2.8 – 51.6)	0.08
rs72520913	*MMP3*	*A/A*	5.0 (-5.7 – 15.7)	0.36	15.6 (1.8 – 29.4)	0.03

* Single nucleotide polymorphism; † mean difference in pain and disability scores for two rare alleles; ∞ 95% confidence interval.

§ COX2 SNP rs4140564 was left out of the analysis due to no G/G genotype in the cohort.

No significant effect of the other covariates included in the models (age, gender and fusion) was observed.

Table 3 describes the study population characteristics and values for post treatment disability and pain reduction according to the genotype for associated SNPs. For *IL18RAP* SNP rs917997, we observed that improvement in disability was reduced among *T/T* homozygotes (*N* = 7, mean [SD] – 2.0 [17.8]) compared to the *C/C* homozygotes (*N* = 50, Mean [SD] – 21.5 [19.6]), and *T/C* heterozygotes (*N* = 36, mean [SD] – 21.4 [21.5]). The improvement in disability for the other associated *IL18RAP* SNP rs1420106 was also the less among *A/A* homozygotes (*N* = 7, mean [SD] – 2.0 [17.8]), where as larger improvement was observed for *A/G* heterozygotes (*N* = 33, mean [SD] – 23.8 [20.8]) and *G/G* homozygotes (*N* = 53, mean [SD] – 20.0 [20.8]).

**Table 3:** **Characteristics of the patients (*N *=93) carrying different genotypes for the associated SNPs**

	***IL18RAP *(rs1420106)**			***IL18RAP *(rs917997)**			***MMP3 *(rs72520913)**		
**Genotype**	***A/A***	***A/G***	***G/G***	***T/T***	***C/T***	***C/C***	***A/A***	***A/C***	***C/C***
Number of Patients	7	33	53	7	36	50	18	56	19
Males / Females	2/5	10/23	23/30	2/5	11/25	22/28	8/10	19/37	8/11
Age at follow-up	49.9 [9.1]	49.3 [12.9]	53.8 [7.3]	49.9 [9.1]	49.8 [8.2]	53.7 [7.4]	51.6 [8.7]	53.2 [6.7]	48.4 [10.0]
Mean [SD]									
Fusion/cognitive	5/2	23/10	32/21	5/2	24/12	31/19	10/8	39/17	11/8
Baseline ODI	43.1 [8.9]	47.6 [12.9]	42.0 [9.6]	43.1 [8.9]	46.5 [18.2]	42.5 [9.6]	40.3 [10.0]	45.1 [11.0]	44.6 [11.8]
Mean [SD]									
Follow-up ODI	41.1 [20.6]	23.9 [18.5]	21.9 [17.6]	41.1 [20.6]	25.1 [18.2]	21.0 [17.6]	25.2 [14.1]	24.6 [19.6]	21.3 [19.8]
Mean [SD]									
ODI change	- 2 [17.8]	- 23.8 [20.8]	- 20.0 [20.8]	- 2 [17.8]	- 21.4 [21.5]	- 21.5 [19.6]	- 15.2 [16.3]	- 20.4 [22.0]	- 23.3 [20.8]
Mean [SD]									
Baseline VAS LBP	56.5 [14.2]	64.5 [13.3]	61.9 [13.7]	56.5 [14.2]	63.7 [13.1]	62.3 [14.0]	59.5 [11.7]	62.5 [13.5]	64.7 [15.9]
Mean [SD]									
Follow-up VAS LBP	48.8 [23.2]	38.4 [25.3]	36.0 [25.8]	48.8 [23.2]	39.1 [24.5]	35.3 [26.3]	48.7 [20.0]	33.8 [25.6]	39.2 [27.5]
Mean [SD]									
VAS LBP change	- 7.7 [24.3]	- 26.1 [25.8]	- 25.9 [32.9]	- 7.7 [24.3]	- 24.6 [25.3]	- 27.0 [33.5]	- 10.8 [18.5]	- 28.7 [33.2]	- 25.5 [26.2]
Mean [SD]									

Worst possible score for ODI and VAS LBP was 100.

Similarly, lower reduction in pain was observed among patients who were homozygous *A/A* for *MMP3* SNP rs72520913 (*N* = 18, mean [SD] – 10.8 [18.5]) compared to the heterozygous *A/C* (*N* = 56, mean [SD] – 28.7 [33.2]) and homozygous *C/C* (*N* = 19, mean [SD] – 25.5 [26.2]) genotypes.

Additive and dominant genetic model association testing revealed no statistically significant associations between post treatment ODI and VAS LBP change and any of the tested markers.

### Associations with severity of degeneration (MRI and CT) and number of discs involved

We observed an association of the *IL18RAP* polymorphism rs1420100 with severe degeneration (*p* = 0.05; odds ratio = 3.9; 95% CI = 1.1-19.3) compared to moderate, and with degeneration of more than one disc compared to one (*p* = 0.02; odds ratio = 4.1; 95% CI = 1.3-14.0), using a dominant model. *MMP3* polymorphism rs72520913 was found to be associated with severe degeneration (*p* = 0.006; odds ratio = 3.5; 95% CI = 1.5-9.4) using the additive model but no associations were observed for the recessive genetic model.

### Haplotype association analysis for severity of degeneration and treatment outcome

The five SNPs (rs2071375, rs2287037, rs1420100, rs1420106, rs917997) from the biologically similar pro-inflammatory neighbouring genes located on chromosome 2 (*IL1A, IL18R1, IL18RAP*) were in LD and four common haplotypes accounted for > 80% of all haplotypes (Table 4).

**Table 4:** **Estimated haplotype frequencies for SNPs at *IL1A, IL18R1 and IL18RAP *chromosome 2 in 93 LDD patients with LBP**

**rs2071375**	**rs2287037**	**rs1420100**	**rs1420106**	**rs917997**	**Frequency %**
C	C	C	A	T	21.2
C	T	A	G	C	7.0
C	T	C	G	C	16.2
T	C	A	G	C	13.6
T	C	C	A	T	4.0
T	T	C	G	C	6.9
*C	C	A	G	C	29.4

* Reference haplotype (most frequent).

Haplotype association analysis revealed significant associations for the haplotype *T-C-C-A-T* with improvement in disability (*p* = 0.02; mean difference = 20.0; 95% CI = 3.0-37.0) and reduction in pain (*p* = 0.04; mean difference = 24.0; 95% CI = 1.4-46.5) at 9 year follow-up. No association of any haplotype of chromosome 2 markers was observed with the severity of degeneration and number of degenerated discs.

## Discussion

Several studies have previously reported the association of inflammatory and matrix degrading genes with LDD [9,10,14], and findings of the present study confirms that some of these genes influence the radiographic degeneration of the lumbar disc. The novelty of the present study is that it also highlights the influence of these variants on pain and disability at long term in patients treated with spinal fusion or cognitive intervention and exercises for CLBP.

This study observed associations of *IL18RAP* polymorphism rs1420100 and *MMP3* polymorphism rs72520913 with severe degeneration and for rs1420100 also with more than one degenerated disc. Interestingly two other polymorphisms of *IL18RAP*, rs917997 and rs1420106, showed association with improvement in disability. Furthermore the *MMP3* polymorphism, rs72520913, was observed to be associated with reduction in pain.

The associations between *IL18RAP* polymorphism rs1420100 with severity of degeneration and the number of degenerated discs are in line with results from a previous study by Videman et al. They were the first to identify an association of this SNP of *IL18RAP* gene with LDD [10]. In their study of 588 monozygotic and dizygotic male twins from the Finish population, they reported a significant association with disc desiccation at both upper and lower lumbar levels, which is considered to be an earlier stage of degenerative process. They did not find an association with disc height narrowing or bulging. In contrast, we only assessed the lower lumbar segments (L4-L5 and L5-S1). These are usually more prone to severe degeneration and our association was observed in patients who had degeneration and chronic LBP and were considered for lumbar fusion. Although our findings strengthen the evidence in support of previously reported association of IL18RAP gene variant with radiographic lumbar disc degeneration, we could not rule out that these findings could be by chance. The small sample size and no correction for multiple testing, does not allow drawing firm conclusions.

IL18RAP is a subunit of IL18 receptor along with IL18R1 [40] and is essential for IL18 signal transduction and ligand binding affinity. IL18R mediated activation of T cells and natural killer cells results in secretion of interferon gamma (IFN-γ), which induce macrophages to secrete cytokines TNF-α and IL-1, which are eventually involved in increased matrix degradation, by production of cytokines and proteases. These have been found to be secreted by the cells of degenerated and herniated discs [41-43]. This sheds light on a putative connection between inflammation and degeneration and a possible pathway for back pain.

Interestingly in the present study, two other tested *IL18RAP* polymorphisms, rs917997 and rs1420106, did not display association with severity of degeneration, but with reduction in disability. The reduction was less pronounced among *T/T* genotypes at rs917997 compared to *C/C* and *C/T* indicating that carrying even one copy of *T* allele was associated with reduced improvement in disability. Similar lower reduction appeared to be attributed to *A* allele for rs1420106 where less improvement was observed with *A/A* compared to the *G/G* and *A/G* genotypes. The alleles at these polymorphisms rs1420106-*A* and rs917997-*T* were in strong LD and both revealed similar association with improvement in disability. On the other hand, the alleles rs1420100-*A* and rs917997-*T* or rs1420106-*A*, were never carried on the same haplotype, but were in complete negative LD (*D'* = - 1). Therefore we speculate that different genetic variants at the same gene, i.e. *IL18RAP*, affect both the severity of degeneration and improvement in outcome. The polymorphisms rs1420106 and rs917997 were found to be associated with disc desiccation in the study by Videman et al, but no association of these polymorphisms was observed with severity of degeneration in the present study. The SNP rs917997 has previously been reported to be associated with differential expression of IL18RAP gene in coeliac disease patients [44].

We observed that a haplotype *T-C-C-A-T* spanning the *IL1A, IL18R1* and *IL18RAP* genes located on chromosome 2, revealed association with improvements in pain and disability. IL-18 is a member of the IL-1 family of cytokines. These are related in terms of structure, receptor family and signal transduction pathways [45] and both are located on chromosome 2. Based on these similarities, we considered the *IL1A* rs2071375 along with *IL18* receptor polymorphisms when testing the association of haplotypes.

The association of *MMP3* SNP rs72520913 with severe degeneration is in agreement with previously reported associations with disc degeneration [9]. This SNP has been reported to regulate the *MMP3* gene expression where the 5A allele possesses twice the promoter activity compared to the 6A allele [25]. A study involving elderly patients found the *5A/5A* genotype to be associated with presence of osteophytes, narrowing of discs and end plate sclerosis [15]. Another study evaluated the interaction between this polymorphism and whole body vibration (gene-environment interaction) and revealed susceptibility between the *5A* allele and LDD [16]. Together with IL1A it has been reported to be associated with type II Modic changes [13]. In vitro studies have also highlighted the role of MMP3 in intervertebral disc degeneration through matrix degradation [46]. Our finding supports the role of variant(s) in promoter region of *MMP3* gene in LDD, and based on the fact that such polymorphisms may enhance the MMP3 expression, we speculate that they may ultimately disturb the balance of ECM of the lumbar disc. Contrary to this, the association of the same polymorphism with improvement in pain after treatment, is difficult to understand, but in line with other studies that have reported a low correlation between clinical and radiographic outcome [12,32].

The inflammatory genes tested in the present study are interestingly also involved in the modulation of pain. They regulate the production of cytokines that may contribute to LBP by inducing growth of nerve and vascular elements [47,48]. IL18 induced synthesis of tumor necrosis factor-alpha (TNFα) has also been proposed to play a role in discogenic pain [49]. MMP3 and IL1A have been associated with neuropathic pain and sensitization of nocireceptors [18,24].

Our finding of a higher yield of DNA extracted from saliva compared to blood was similar to Mitsouras et al report that DNA yield from saliva was superior to blood and buccal swabs [50]. In accordance with our finding that saliva DNA was of high quality, a study had previously reported the saliva DNA extracted using similar kits and methods as ours, to be of high quality [51]. Other studies have reported an equal or lower DNA yield from saliva compared to the blood [28,52].

The main strengths of the present study include long term follow up of patients, a high genotype success rate alongside homogeneity of the sample population (all subjects were of homogenous Norwegian origin) and testing of polymorphisms from genes that have previously been reported to be involved in both degeneration and pain modulation.

Limitations were small sample size, no testing for multiple correction, dichotomy of images of degeneration into moderate and severe rather than using a continuous classification, inclusion of patients with only 1-2 levels involved rather than multi level degeneration and testing by three genetic models on the same data. Furthermore, we have only tested one or few variants from each of these inflammatory genes, thus we could have missed the involvement of genetic variation at each tested gene.

Despite these limitations, our study gives valuable replication of previous findings, support the importance of variation at inflammatory and matrix degrading genes in the degeneration process and LBP and provide hypothesis for future studies. Replication of these results in larger study populations is important for strengthening conclusions from the present study.

## Conclusions

This study suggests a role of variation at inflammatory and matrix degrading genes in severity of LDD and pain and disability outcome.

## Abbreviations

LDD: Lumbar disc degeneration; CLBP: Chronic low back pain; LBP: Low back pain; SNPs: Single-nucleotide polymorphisms; ODI: Oswestry disability index; VAS: Visual analog score; ECM: Extracellular matrix; MMP: Matrix metalloproteinase; LD: Linkage disequilibrium.

## Competing interest

The authors declare that they have no competing interests.

## Authors’ contributions

AO, BL, OR and JIB contributed to the conception and design and conduction of the study. JIB recruited and examined the patients, provided funding and approvals. MH, AO and BL conducted the statistical analysis. AO drafted the manuscript and all authors critically revised the manuscript and approved the final version.

## Pre-publication history

The pre-publication history for this paper can be accessed here:

http://www.biomedcentral.com/1471-2474/14/105/prepub
